# Biopsia líquida: Un paso adelante en la medicina de laboratorio

**DOI:** 10.1515/almed-2025-0117

**Published:** 2025-08-20

**Authors:** Angel Díaz-Lagares, Wladimiro Jiménez

**Affiliations:** Epigenomics Unit, Cancer Epigenomics, Translational Medical Oncology Group (ONCOMET), Health Research Institute of Santiago de Compostela (IDIS), University Clinical Hospital of Santiago (CHUS/SERGAS), Santiago de Compostela, España; Centro de Investigación Biomédica en Red Cáncer (CIBERONC), ISCIII, Madrid, España; Department of Clinical Analysis, University Hospital Complex of Santiago de Compostela (CHUS), Santiago de Compostela, España; Servicio Bioquímica i Gen. Mol, Catedrático Bioquímica, Hospital Clinic / Fac. Medicina, Universitat Barcelona, España

En este número de *Avances en Medicina de Laboratorio* presentamos un monográfico sobre la biopsia líquida, un método diagnóstico no invasivo llamado a transformar la práctica habitual en nuestros laboratorios clínicos. La biopsia líquida permite analizar el ADN tumoral circulante (ctDNA, por sus siglas en inglés), las células tumorales circulantes (CTCs) o la presencia de vesículas extracelulares (exosomas) en fluidos corporales como la sangre, la orina o el líquido cefalorraquídeo. La biopsia líquida se postula como un método más seguro, reproducible y dinámico de realizar en el seguimiento prenatal de aneuploidías fetales y otras alteraciones durante la gestación, así como monitorizar la evolución tumoral, la respuesta a tratamiento, o los mecanismos de resistencia [1]. Aunque este procedimiento no invasivo se empezó a utilizar ya en la década de los 90 para la detección de mutaciones tumorales en el ctDNA, el término “biopsia líquida” no se estableció formalmente hasta 2010 [2].

Posteriormente, durante la primera década del presente siglo, las aplicaciones de la biopsia líquida se multiplicaron con el desarrollo de nuevas técnicas de alto rendimiento y elevada sensibilidad, como la PCR digital y la secuenciación de nueva generación (NGS). La primera prueba de biopsia líquida (Roche Cobas EGFR Mutation test) fue aprobada por la US Federal Drug Administration (FDA) en 2016 para la detección de mutaciones en *EGFR* en el carcinoma pulmonar no microcítico, lo cual representó todo un hito, ya que con ello se confirmaba la utilidad clínica de la biopsia líquida en la oncología diagnóstica. La adopción de la biopsia líquida en los laboratorios clínicos ha sido posible gracias a diversos avances tecnológicos. En primer lugar, las técnicas de NGS permiten secuenciar millones de fragmentos de ADN, permitiendo la detección de mutaciones de baja frecuencia en ctDNA. En segundo lugar, la PCR digital, con elevada sensibilidad para la detección de mutaciones específicas, permite realizar una cuantificación precisa de alelos mutantes de baja frecuencia. Por otro lado, las plataformas microfluídicas han mejorado notablemente el aislamiento y análisis de CTC infrecuentes en muestras de sangre. Por su parte, los exosomas, esto es, pequeñas vesículas extracelulares secretadas por las células tumorales que contienen ADN, ARN y proteínas, suponen otra fuente fiable de biomarcadores tumorales. Finalmente, los avances en bioinformática e inteligencia artificial (IA) han facilitado la interpretación de datos genómicos complejos.

La biopsia líquida ofrece diversas ventajas que justifican su aplicación en la clínica. Al tratarse de un método no invasivo, se elimina la necesidad de realizar procedimientos quirúrgicos, reduciendo de este modo las molestias al paciente y los riesgos inherentes a toda cirugía. Esta técnica resulta de especial utilidad en pacientes con enfermedad avanzada, un mal estado funcional, o que presentan tumores en zonas de difícil acceso. Así mismo, la facilidad para obtener muestras permite repetir el análisis en diferentes puntos temporales, lo que resulta inviable con las biopsias de tejido. Del mismo modo, a diferencia de la biopsia de tejido, que ofrece una instantánea de una sola parte del tumor, la biopsia líquida refleja la heterogeneidad espacial y temporal del tumor. Así, los analitos producidos por las células tumorales en el torrente sanguíneo proceden de múltiples focos tumorales, lo que permite obtener un perfil molecular más completo de la enfermedad [3].

La especialidad en la que el uso de la biopsia líquida está más extendido es en el de la oncología, ya que el análisis del ctDNA permite detectar mutaciones somáticas, fusiones de genes, cambios epigenéticos, y variaciones en el número de copias, proporcionando así una representación de la carga tumoral y la heterogeneidad molecular del tumor. En la práctica clínica, la utilidad del análisis de ctDNA ha quedado demostrada en diversos escenarios, especialmente cuando se dispone de una cantidad limitada de tejido o el tumor se encuentra en una zona inaccesible, permitiendo, por ejemplo, la detección temprana del cáncer, la selección de terapias dirigidas, la detección de mutaciones de resistencia durante la administración de sucesivas líneas de tratamiento, el seguimiento de la respuesta al tratamiento, la detección de (enfermedad mínima residual) EMR o la detección precoz de recurrencias, que a menudo preceden a los hallazgos en los estudios de imagen. Cabe destacar que dicha utilidad clínica se ha demostrado en una amplia variedad de enfermedades oncológicas, como el cáncer de pulmón, mama, colon, páncreas y próstata, por mencionar solo algunos. Por otro lado, se están desarrollando paneles multigénicos para distintos tipos de neoplasias, así como estrategias agnósticas al tipo de tumor, lo que permite realizar una caracterización genómica y epigenómica completa a partir del análisis del ADN libre circulante (cfDNA, por sus siglas en inglés) [4].

El carácter revolucionario de la biopsia líquida radica en su capacidad para facilitar el diseño de tratamientos personalizados. Al capturar la evolución del tumor y su dinámica clonal, se pueden adaptar las terapias partiendo de datos moleculares obtenidos en tiempo real. Este seguimiento dinámico resulta crucial en contextos como la inmunoterapia, donde la identificación precoz de los pacientes que no responden a tratamiento o que presentan hiperprogresión permite ajustar el tratamiento de forma inmediata. Por otro lado, con la biopsia líquida se puede realizar una vigilancia longitudinal de la enfermedad, lo que resulta crucial a la hora de detectar EMR o recurrencia temprana en pacientes en remisión. En dicho contexto, la biopsia líquida se emplea como centinela molecular, al poder detectar recurrencias semanas o meses antes de ser visibles en los estudios de imagen, facilitando de este modo la intervención precoz [2].

Las aplicaciones de la biopsia líquida se extienden más allá del campo de la oncología, ya que en la medicina prenatal, por ejemplo, el cfDNA de plasma materno se está empleando en el cribado prenatal no invasivo (NIPT), permitiendo la detección de aneuploidías fetales con una elevada precisión y un riesgo mínimo. En el ámbito de los trasplantes, el cfDNA del donante (dd-cfDNA) se utiliza como marcador precoz de rechazo del órgano, ofreciendo información muy valiosa con anterioridad a que se produzca el deterioro clínico o se evidencien cambios histológicos. En las enfermedades infecciosas, la secuenciación de cfDNA permite detectar directamente en el plasma la presencia de patógenos, lo que resulta de especial utilidad en los pacientes críticos, o cuando el resultado de los cultivos es negativo. Así mismo, se están llevando a cabo estudios para investigar la función del cfDNA y otros analitos de biopsia líquida en las enfermedades autoinmunes, enfermedades cardiovasculares, alteraciones endocrinas y trastornos neurodegenerativos en los que el daño y la remodelación celular pueden dejar una huella molecular en la circulación.

Este panorama alentador también presenta importantes limitaciones técnicas y clínicas, entre las que destacan aquellas relacionadas con la limitada sensibilidad del método. De hecho, la reducida presencia de ctDNA en los primeros estadios del cáncer o en tumores con una baja tasa de secreción puede afectar a la fiabilidad de los resultados. En estos casos, el ctDNA puede llegar a constituir menos del 0,01 % del cfDNA, lo que complica extremadamente su detección, pudiendo derivar en falsos negativos, especialmente en la detección precoz o en el seguimiento de la EMR. Además, nos podemos encontrar con falta de especificidad, como es el caso de los falsos positivos en la hematopoyesis clonal de potencial indeterminado (CHIP). En dichos casos, las células sanguíneas adquieren mutaciones somáticas no relacionadas con el cáncer que pueden ser interpretadas erróneamente como de origen tumoral, con la consiguiente toma de decisiones inadecuadas. Otra limitación es la ausencia de un protocolo estandarizado universal para la obtención, el procesamiento y el análisis de muestras de biopsia líquida. La variabilidad de los procedimientos preanalíticos y analíticos puede afectar a la reproducibilidad de los mismos y dificultar la comparación de resultados entre laboratorios [5].

Pese a los avances tecnológicos logrados, el análisis de biopsia líquida mediante NGS sigue presentando un elevado coste, lo que puede limitar el acceso al mismo en ciertos contextos sanitarios. Las políticas de cobertura sanitaria, las aprobaciones regulatorias y las guías clínicas están en constante evolución, lo que provoca una adopción heterogénea entre países e instituciones. En este contexto, la estandarización de los procedimientos de biopsia líquida, sumada a la adherencia a los programas de garantía de la calidad, resultan esenciales a la hora de garantizar la aplicación de buenas prácticas y la obtención de resultados fiables, y consolidar así la confianza del personal clínico. Además, el número de pruebas de biopsia líquida que se han sometido a un proceso riguroso de validación clínica sigue siendo limitado, por lo que es necesario realizar estudios prospectivos a gran escala para determinar su utilidad en los diferentes tipos y estadios del cáncer.

El campo de la biopsia líquida está experimentando un rápido y notable crecimiento. Tecnologías emergentes como las llamadas “ómicas” (p.ej. epigenómica y fragmentómica), unidas a los algoritmos de aprendizaje automático y los ensayos multianalito, están llamadas a mejorar el rendimiento diagnóstico y ampliar las aplicaciones clínicas de la biopsia líquida. En la medicina de laboratorio, esta técnica ofrece una oportunidad única de liderar la innovación en el lugar intermedio entre el diagnóstico molecular y la asistencia médica. Con la adopción de esta tecnología, los laboratorios clínicos contribuirán a establecer un diagnóstico temprano, mejorar los resultados clínicos de los pacientes, y ofrecer una medicina más personalizada.

En conclusión, la biopsia líquida no supone únicamente una innovación tecnológica, sino que posee un poder transformador que va a revolucionar el conocimiento y el manejo de las enfermedades. Pese a sus actuales limitaciones, la implementación de esta técnica en la práctica rutinaria de laboratorio supone un paso adelante en la evolución de la medicina de laboratorio.
